# Optimization and Prediction of the Drying and Quality of Turnip Slices by Convective-Infrared Dryer under Various Pretreatments by RSM and ANFIS Methods

**DOI:** 10.3390/foods10020284

**Published:** 2021-01-31

**Authors:** Ebrahim Taghinezhad, Mohammad Kaveh, Antoni Szumny

**Affiliations:** 1 Department of Agricultural Technology Engineering, Moghan College of Agriculture and Natural Resources, University of Mohaghegh Ardabili, Ardabil 56199-11367, Iran; 2 Faculty of Agriculture and Natural Resources, University of Mohaghegh Ardabili, Ardabil 56199-11367, Iran; sirwankaweh@uma.ac.ir; 3 Department of Chemistry, Wroclaw University of Environmental and Life Science, CK Norwida 25, 50-375 Wrocław, Poland; antoni.szumny@upwr.edu.pl

**Keywords:** blanching, drying, efficiency, energy, microwave, ultrasound

## Abstract

Drying can prolong the shelf life of a product by reducing microbial activities while facilitating its transportation and storage by decreasing the product weight and volume. The quality factors of the drying process are among the important issues in the drying of food and agricultural products. In this study, the effects of several independent variables such as the temperature of the drying air (50, 60, and 70 °C) and the thickness of the samples (2, 4, and 6 mm) were studied on the response variables including the quality indices (color difference and shrinkage) and drying factors (drying time, effective moisture diffusivity coefficient, specific energy consumption (*SEC*), energy efficiency and dryer efficiency) of the turnip slices dried by a hybrid convective-infrared (HCIR) dryer. Before drying, the samples were treated by three pretreatments: microwave (360 W for 2.5 min), ultrasonic (at 30 °C for 10 min) and blanching (at 90 °C for 2 min). The statistical analyses of the data and optimization of the drying process were achieved by the response surface method (RSM) and the response variables were predicted by the adaptive neuro-fuzzy inference system (ANFIS) model. The results indicated that an increase in the dryer temperature and a decline in the thickness of the sample can enhance the evaporation rate of the samples which will decrease the drying time (40–20 min), *SEC* (from 168.98 to 21.57 MJ/kg), color difference (from 50.59 to 15.38) and shrinkage (from 67.84% to 24.28%) while increasing the effective moisture diffusivity coefficient (from 1.007 × 10^−9^ to 8.11 × 10^−9^ m^2^/s), energy efficiency (from 0.89% to 15.23%) and dryer efficiency (from 2.11% to 21.2%). Compared to ultrasonic and blanching, microwave pretreatment increased the energy and drying efficiency; while the variations in the color and shrinkage were the lowest in the ultrasonic pretreatment. The optimal condition involved the temperature of 70 °C and sample thickness of 2 mm with the desirability above 0.89. The ANFIS model also managed to predict the response variables with *R*^2^ > 0.96.

## 1. Introduction

The turnip has been long used in the human diet due to its high vitamin and mineral contents. Its use dates back to the prehistoric era. The turnip is cultivated in Europe and Iran, especially in cold regions [1]. Recently, the turnip has attracted the attention of consumers due to its high antioxidant content and anti-inflammatory, anti-diabetes, and anticancer features, in addition to its glucosinolates, flavonoids, and phenylpropanoid contents [2].

During the drying process, the moisture content of the product will be declined by heat and simultaneous mass transfer between the surroundings and sample surfaces. This process can be used as one of the important storage methods to prolong the shelf life of the product, reduce its transportation costs and minimize its packing requirements [3]. Drying can also prevent the spoilage and wastes of the crops after their harvest [4].

Among various industrial commercial dryers, convective dryers have found extensive applications in diverse industries including food and agriculture. This method, however, suffers from serious problems such as long processing time, low efficiency, high energy consumption rate, and declining quality of the product [5]. To resolve these issues, novel technologies such as hybrid dryers with the use of pretreatments can be employed [6].

The infrared method is one of the recent approaches used in the drying of food products, this method often applied in combination with convective methods and its goal is to accelerate the process of drying, reduce the energy consumption and improve the quality of the final product [7]; for instance, a hybrid convective-infrared (HCIR) dryer was used to dry blackberry [7,8] and potato [9]. Today, various pretreatments have been employed to reduce the drying time and improve the quality of the crops. Using these pretreatments, it is possible to reduce some of the unwanted variations such as textural and color changes [10]. So far, various pretreatments have been employed in the drying industry. Ultrasound and blanching pretreatments were used in a hybrid microwave-convective dryer to dry parsley leaves [11]. In another study, osmotic and ultrasound pretreatment were employed for drying strawberries under convective drying [12]. Ethanol and ultrasound were used as a pretreatment to dry potatoes using an infrared (IR) drying approach [13], citric acid and blanching were used to dry cauliflower using the convective dryer [10]. Other studies have been conducted by various dryers using different pretreatments to dry diverse crops for instance, blackberry [14], raspberries [15], Mirabelle plum [16], cranberry snacks [17], carrot discs [5], and cabbage [18]. These studies indicated that the use of these pretreatments can increase the effective moisture diffusion coefficient while reducing the drying time and specific energy consumption (*SEC*); hence improving the quality of the dried products. To the best of our knowledge, no study has addressed the influence of various pretreatments on the drying process of turnip slices using a hybrid convective-infrared (HCIR) dryer.

The relationship between the independent and dependent variables of the drying process is of crucial significance. Although some of the numerical methods have managed to some extent to resolve the complexity of the non-linear behavior. Due to the limitations of these methods, researchers have focused on other statistical methods such as adaptive neuro-fuzzy inference system (ANFIS) and response surface methods [19]. The neural-fuzzy deductive systems simultaneously exploit the merits of the artificial neural network and fuzzy logic. This method can be used to approximate the non-linear relationship between the inputs and outputs and has shown bright capabilities in the training, construction, and classification stages [20]. The response surface method (RSM) is a series of mathematical and statistical methods to model and analyze problems in which the response variable is under the influence of several independent variables. This method is aimed to optimize the response variables [21]. In RSM optimization, input variables are defined as the independent ones and their influence on the response (dependent) variable is explored. Numerous researchers have used RSM and ANFIS to model and optimize the quality and drying process of various crops including okra [22], quince [23], yacon [24], lavender leaves [25], and rough rice [26] for RSM and blackberries [8], almond [20], and yam slices [27] using the ANFIS method.

Regarding the importance of turnip in the human diet, its storage at high quality is of crucial significance. Previous studies have shown that no work has addressed the use of RSM and ANFIS to optimize and predict the quality and drying process of the turnip slices using HCIR dryers. In this regard, the aim of the present study is to model and optimize the effects of independent variables (slice thickness, temperature) on the dependent variables (drying time, effective moisture diffusion coefficient, *SEC*, energy efficiency, drying efficiency, shrinkage, and color) in drying turnip slices. In this research, turnip slices at the thicknesses of 2, 4, and 6 mm were dried by an HCIR dryer after various pretreatments (microwave, ultrasound, and blanching).

## 2. Materials and Methods

### 2.1. Turnip Preparation

Fresh turnips were provided from ParsAbad City, Ardebil province (Iran). The samples were kept in a refrigerator at + 4 °C. Prior to the experiments, the turnip samples were left at room temperature for 1 h. The initial moisture of the samples was determined at 10.23% (d.b.) using an oven (Memmert company, UFB50 model, Schwabach Germany) at 70 °C for 24 h.

### 2.2. Pretreatments

The pretreatments were carried out on the turnip samples before the drying process as follows:

#### 2.2.1. Blanching

For blanching pretreatment, a warm water bath (Memmert, WNB 14, Schwabach, Germany) was used. This bath had a maximum temperature of 120 °C and an accuracy of ±0.1 °C. The samples were placed in a warm bath at 90 °C for 2 min [17].

#### 2.2.2. Ultrasound Pre-Treatment

Ultrasound pretreatment was carried out using an ultrasonic bath (Parsonic, 7500s, Tehran, Iran) at the frequency of 28 kHz and power of 70 W regarding the constant frequency of the bath, turnip samples were immersed in distilled water at 30 °C and exposed to ultrasound waves for 10 min [28].

#### 2.2.3. Microwave Pre-Treatment

A domestic microwave oven (Panasonic NN-C2002W, Tokyo, Japan) at the frequency of 50 Hz and maximum heating power of 1000 W (with the capability of tuning the power at 90, 180, 360, 600, and 900 W) was employed for microwave pretreatment of the samples, the pretreatment was carried out at the power of 360 W for 2.5 min [29].

### 2.3. Hybrid Convective-Infrared (HCIR) Dryer

After pretreatments, the drying process was conducted using an HCIR dryer (GC 400 model, company Grouc, Tehran, Iran). This dryer includes two Infrared (IR) lamps (Philips model, Flemish, Belgium) working at the power of 500 W which are installed at the upper part of the drying chamber at the height of 30 cm the dryer has a centrifuge blower to blow hot air parallel to the substrate. To create the input air, a centrifuge fan equipped with an inverter (Vincker VSD2, ABB Co., Taipei, Taiwan) was employed. The input air speed was set at 1 m/s. The samples were placed on a meshed container on a digital balance (AND, GF-6000, A&D Company Ltd., Tokyo, Japan) at the accuracy of 0.01 g placed beneath the channel. The turnips were cut into 2, 4 and 6 mm thick pieces and pretreated using blanching, microwave and ultrasonic methods. The samples were then dried with a HCIR dryer at three temperature levels (50, 60 and 70 °C). Before the tests, the dryer was operated for 15 min to reach a constant temperature and air speed. In each test, one layer of 40 g turnip slice was placed on the dryer tray. During the drying process, the mean temperature and air humidity were 20 ± 4 °C and 15 ± 5%, respectively.

### 2.4. Moisture Ratio

Moisture ratio of the hybrid convective-infrared (HCIR)-dried turnip slices was determined by Equation (1) [1]:

(1) $$MR=\frac{M_{t}-M_{e}}{M_{b}-M_{e}}$$

### 2.5. Effective Moisture Diffusivity

Fick’s law, Equation (2), can describe the moisture transport in the descending stage of the drying process [30]:

(2) $$\frac{\partial X}{\partial t}=D_{eff}\frac{\partial^{2}X}{\partial x^{2}}$$

The second Fick’s law is related to the mass diffusivity during the descending phase of the drying process, using appropriate boundary conditions, it is possible to solve the Fick’s equation for various geometries. For a thin layer, the Fick equation can be solved by Equation (3) [31]:

(3) $$MR=\frac{8}{\pi^{2}}\sum_{n=1}^{\infty }\frac{1}{\left(2n+1\right)}\exp\left(\frac{-D_{eff}{\left(2n+1\right)}^{2}\pi^{2}t}{4L^{2}}\right)$$

The effective diffusivity coefficient can be determined from the slope of Equation (4) [17]:

(4) $$\ln(MR)=\ln(\frac{8}{\pi^{2}})-\ln\left(\frac{-D_{eff}\pi^{2}t}{4L^{2}}\right)$$

Generally, the diffusivity coefficient can be determined by plotting the experimental data of ln(*MR*) versus the time. The slope of the obtained line can be substituted in Equation (5) to determine the diffusivity coefficient [28]:

(5) $$K=\left(\frac{D_{eff}\pi^{2}}{4L^{2}}\right)$$

### 2.6. Specific Energy Consumption (SEC), Energy and Drying Efficiency

After the drying tests, the drying curve and hence the drying time can be determined for each specific condition. The specific energy consumption of the drying process can be obtained by Equation (6) [32]:

(6) $$SEC=\left(\frac{E_{t}}{M_{w}}\right)$$

Energy efficiency can be also determined by Equation (7) [33]:

(7) $$\eta_{e}=\left(\frac{E_{evap}}{E_{t}{}^{}}\right)\times 100$$

The HCIR dryer efficiency can be calculated by the following equation [34]:

(8) $$\eta_{d}=\left(\frac{E_{evap}+E_{heating}}{E_{t}{}^{}}\right)\times 100$$

### 2.7. Shrinkage Measurement

Shrinkage refers to the variations in the sample volume relative to its initial volume. This phenomenon can be assigned to the water removal from the cellular space and its substitution with the air. During the drying process, the shape and size of the product may also change. The alterations in the physical properties can finally result in some changes in the final texture (shrinkage) of the dried products. Shrinkage can be determined by [35]:

(9) $$S_{a}=(1-\frac{V_{t}}{V_{0}})\times 100$$

In which $S_{a}$ shows the shrinkage percentage, *V_t_* denotes the apparent volume of the dried sample (cm^3^) after the time of t and *V*_0_ represents the volume of the raw samples (cm^3^). The apparent volume of the samples was measured by the toluene displacement method using a glass pycnometer (50 mL) in this method, the samples with determined weight were transferred into a semi-filled pycnometer containing toluene. The remaining volume of the pycnometer was then closely filled with the solvent and its weight was measured. The apparent volume of the samples (*V*) can be determined by the following equations [36]:

(10) $$V=V_{f}-\frac{M_{sf}}{\rho_{s}}$$

(11) $$M_{sf}=M_{t+s}-M_{f}-M$$

### 2.8. Color Difference

Color is a significant factor in the evaluation of food products and their marketability [37]. To evaluate the color of the samples, a color meter was used to measure various parameters including *L* (lightness), *a* (red-green), and *b* (yellow-blue). The total color difference of the samples was also determined by Equation (12) [5].

(12) $$\Delta E=\sqrt{{\left(L-L_{0}^{*}\right)}^{2}+{\left(a-a_{0}^{*}\right)}^{2}+{\left(b-b_{0}^{*}\right)}^{2}}$$

### 2.9. Response Surface Methodology (RSM)

In this research, the influence of the independent variables (drying air temperature in three levels of 50, 60, and 70 and the sample thickness in three levels of 2, 4, and 6 mm) on the dependent variables (drying time (min), effective moisture diffusivity (m^2^/s), *SEC* (Mj/kg), energy efficiency (%), drying efficiency (%), shrinkage (%), and color difference) was evaluated for the samples pretreated by microwave, ultrasound, and blanching.

For the predicted responses, it was assumed that:

(13) $$y_{k}=f_{k}(\varepsilon_{1},\varepsilon_{2},\varepsilon_{3})$$

In which *y_k_* is the predicted response and $\varepsilon_{1}$, $\varepsilon_{2}$ and $\varepsilon_{3}$ denote the natural (independent) variables. The second-order response surface equations are also presented in Equation (14) [25]:

(14) $$y_{k}=\beta_{0}+\sum_{j=1}^{k}\beta_{j}x_{j}+\sum_{j=1}^{k}\beta_{jj}x_{j}^{2}+\sum \sum_{i<j}^{k}\beta_{ij}x_{i}x_{j}$$

In the above equation, $\beta_{0}$, $\beta_{j}$, $\beta_{jj}$, and $\beta_{ij}$ are the regression coefficients. *x_j_* also denotes the coded input variables. Design-expert software was used for fitting the response surfaces and optimize the drying process through solving a multiple regression equation (Equation (14)) using historical data and RSM. The mathematical models of each response were assessed by multiple linear regression analysis. The statistical significance of the independent variables for the response variables was explored at the confidence level of 95% (*p* < 0.05). Only the significant variables were included in the proposed regression equation. Finally, the optimal point of the process was determined according to the boundary conditions and the target functions as shown in Table 1.

### 2.10. Adaptive Neuro-Fuzzy Inference System (ANFIS)

Compatible deductive neural-fuzzy systems combine the ANN and fuzzy logic concepts and employ a series of if-then fuzzy laws. In this study, neural-fuzzy modeling was achieved using Matlab software. To this end, a Sugeno system was employed and the desirable membership function was determined among various functions (triangular, trapezoidal, bell-shaped, Gaussian, Pi, type-II Gaussian, and sigmoid). Their membership degree was also obtained by trial and error. A combinational training algorithm (including error back propagations algorithm and minimum square error method) was employed to train and match with the fuzzy deductive system. This model was used to predict the drying time, effective moisture diffusivity coefficient, *SEC*, energy efficiency, drying efficiency, color, and shrinkage of the turnip samples dried under various pretreatment conditions. ANFIS inputs were the input air temperature and the sample thickness. In the present study, 75% of the data were used for training, and the remaining 25% were used for validation. The model evaluation and comparison was carried out by the determination coefficient (*R*^2^), and mean square root error (*MSE*).

(15) $$R^{2}=1-\frac{\sum_{i=1}^{N}{\left(S_{k}-T_{k}\right)}^{2}}{\sum_{i=1}^{N}{\left(S_{k}-T_{m}\right)}^{2}}$$

(16) $$MSE=\frac{1}{N}\sum_{i=1}^{N}{\left(S_{K}-T_{k}\right)}^{2}$$

## 3. Results and Discussion

### 3.1. Drying Time

Table 2 shows the results obtained from the RSM method for predicting the drying time of the turnip slices based on the independent variables (drying air temperature, and slice thickness) for various pretreatments. The drying air temperature and slice thickness had a significant effect on the drying time for all three pretreatments (*p* < 0.05). The fitted models were linear and second-order polynomial equations. The positive and negative signs of the estimated regression in the equations indicated the significant direct and indirect effects on the response variable, respectively (*p* < 0.05).

**Figure 1:**
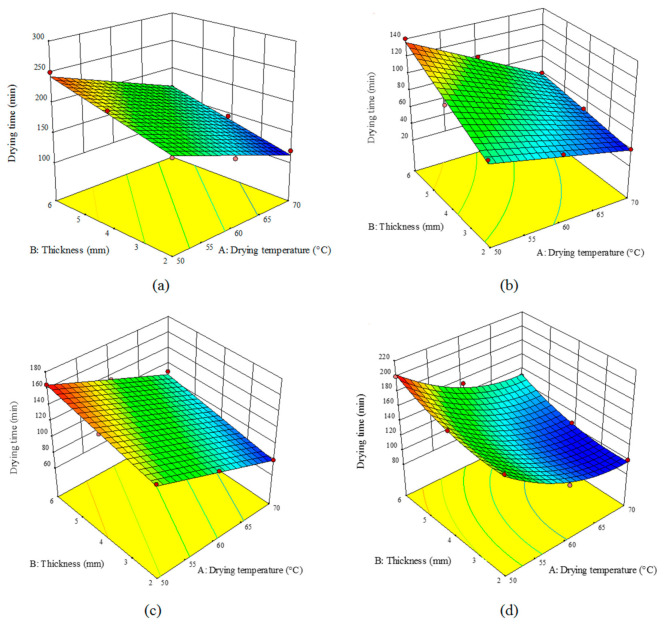
Effect of the drying temperature and sample thickness on the drying time (min) of the turnip slices dried under an hybrid convective-infrared (HCIR) dryer with various pretreatments (**a**) control, (**b**) microwave, (**c**) ultrasound, and (**d**) blanching.

Figure 1, depicts the effect of the drying temperature and slice thickness on the drying time of the turnip samples for the three studied pretreatments using an HCIR dryer. According to Figure 1b, the shortest drying time (40 min) was for the drying air temperature of 70 °C and thickness of 2 mm for the sample pretreated by microwave. The longest drying time (250 min) was also recorded for the drying air temperature of 50 °C for the control samples with the thickness of 6 mm (Figure 1a) the decline in the thickness and the rise in the temperature could enhance the thermal gradient within the turnip samples, hence raising the moisture evaporation rate. The microwave pretreatment also led to a high pressure difference between the center and the surface of the product and incremented the drying rate; this will enhance the mass transfer, hence shortening the drying time [38]. Similar results were reported by the other researchers using a convective dryer and various pretreatments for drying blackberry [39], apple [29], potatoes [31], and black mulberry [7]. According to Figure 1c,d), ultrasound pretreatment also caused a significant (*p* < 0.05) reduction in the drying time, as compared with the blanching pretreatment. The shortest drying time for the ultrasound (140 min) and blanching (170 min) pretreatments were observed in the sample with a thickness of 2 mm dried at the temperature of 70 °C. The ultrasound-induced cavitation can lead to the formation of a series of microchannels in the product which can decrease the boundary layer of the propagation and enhance the mass transfer; this will, in turn, facilitate the water removal from the product [37]. These results are in line with the previous reports. For apple [38] and rose flower [40] drying, the drying time was significantly decreased by ultrasound pretreatment as compared with the blanching pretreatment.

### 3.2. Effective Moisture Diffusivity Coefficient (D_eff_)

Table 3 lists *D_eff_* results for various pretreatments at the studied temperature and thicknesses. *R*^2^ was larger than 0.6 indicating that the demonstrated models were the best models for predicting the value of *D_eff_*. According to Table 3, *D_eff_* showed a linear and significant variation in different pretreatments (*p* < 0.05).

**Figure 2:**
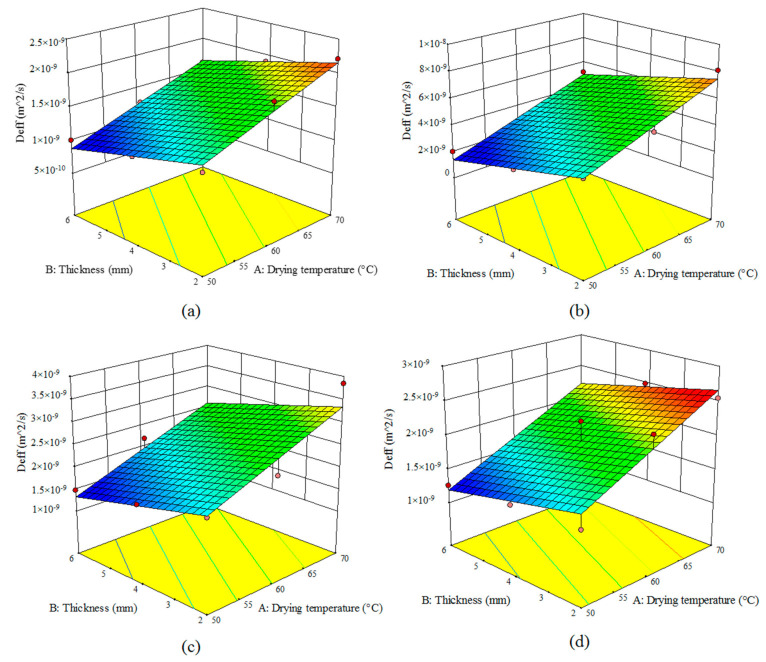
Effect of the drying temperature and sample thickness on the effective moisture diffusivity coefficient (*D_eff_*) (m^2^/s) of the turnip slices dried under an HCIR dryer with various pretreatments (**a**) control, (**b**) microwave, (**c**) ultrasound, and (**d**) blanching.

**Figure 3:**
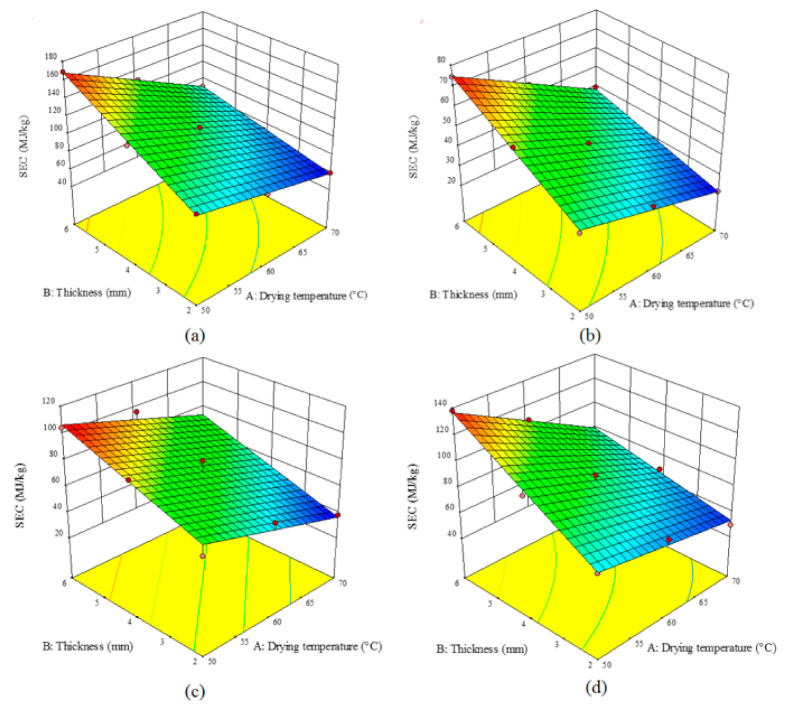
Effect of the drying temperature and sample thickness on the specific energy consumption (*SEC*, MJ/kg) of an HCIR dryer with various pretreatments (**a**) control, (**b**) microwave, (**c**) ultrasound, and (**d**) blanching.

Figure 2 shows the influence of the air temperature and turnip thickness on *D_eff_* for an HCIR dryer with various pretreatments. The highest *D_eff_* value (8.11 × 10^−9^ m^2^/s) was observed for the microwave-pretreated samples dried at the temperature of 70 °C and thickness of 2 mm (Figure 2b); while the lowest *D_eff_* (1.007 × 10^−9^ m^2^/s) was recorded for the control samples with the thickness of 6 mm dried at 50 °C (Figure 2a). Other researchers reported the effective moisture diffusivity in the range of 5.47 × 10^−10^ to 4.82 × 10^−9^ m^2^/s [1,41]. Based on Figure 3, an increase in the input air temperature and a decline in the sample’s thickness can raise *D_eff_*. At high temperatures, the free water of the sample can be evaporated rapidly, hence dramatically reducing the drying time and increasing *D_eff_*. The use of microwave pretreatment is also enhanced, compared to the other pretreatments. By polarizing the water molecules, the microwave increased the internal temperature of the product. Moreover, it destroyed the product texture and formed channels with larger diameters, thus preventing the surface from hardening, hence accelerating the free water evaporation. *D_eff_* will decrease as a result of a decline in the drying time [33]. Similar results were reported by other researchers for cranberry snacks [17], blackberry [30], and okra [42]. They declared that the use of different pretreatments can increase the moisture diffusivity coefficient compared to the control samples.

Based on Figure 2c,d, *D_eff_* was higher in the ultrasonic pretreatment as compared with the blanching as ultrasonic treatment could open capillary paths due to the dispersion of the surface species; giving rise to longer microscopic channels as a result of the deformation of the cell. Therefore, ultrasonic pretreatment can deform and destroy the cell walls and accelerate moisture evaporation [38]. These results are in line with the previous reports by other researchers [30,39].

### 3.3. Specific Energy Consumption (SEC)

Table 4 shows the modeling results for *SEC* of drying turnip slices at various temperatures and sample thicknesses and pretreatments. Based on this table, the linear variables of air temperature and sample thickness could significantly (*p* < 0.05) affect *SEC* in different pretreatments. *R*^2^ was larger than 0.84, indicating the suitability of this linear model for predicting the value of *SEC*. It must be noted that only the coefficients with significant (*p* < 0.05) impact on *SEC* are included in the equation.

Figure 3, depicts the effects of the temperature of the drying air and the sample thickness on the value of *SEC* for various pretreatments. The highest *SEC* (168.98 MJ/kg) was related to the control samples with the thickness of 6 mm dried at 50 °C (Figure 3a); while the lowest *SEC* (21.57 kJ/kg) was observed for the microwave-pretreated samples with the thickness of 2 mm dried at 70 °C (Figure 3b). Similar results were reported by the other researchers in drying black mulberry [7], blackberry [39], and apple [34] using convective dryer under different pretreatments. They indicated that microwave-treated and control samples had the lowest and highest *SEC* values, respectively. In the current study, microwave pretreatment declined the *SEC* compared to the other two pretreatments. Using microwave pretreatments, the destruction in the texture of the product will be enhanced which will elevate the moisture removal rate; hence declining the *SEC* value [32]. Compared to blanching pretreatment, ultrasonic pretreatment led to lower *SEC* values (Figure 3c,d). Food products such as turnip will form a hard layer on their surface following the moisture removal which may decelerate the evaporation. Ultrasonic pretreatment prevents the formation of this layer, hence increasing the moisture removal rate, shortening the drying time, and hence reducing the *SEC* value [20]. Similar results were reported for drying parsley leaves by a microwave-convective dryer [11] and blackberry by an HCIR dryer [43]; as they showed that ultrasound pretreatment can result in lower *SEC* values, compared to the blanching pretreatment.

### 3.4. Energy ($\eta_{e}$) and Dryer ($\eta_{d}$) Efficiency

Table 5 lists the results obtained by modeling the effects of drying air temperature and sample thickness on the energy and dryer efficiency using an HCIR dryer with different pretreatments. Under all the studied conditions, *R*^2^ was above 0.89 for the energy efficiency and above 0.8 for the dryer efficiency indicating that these models can predict the energy and dryer efficiencies well. Under the ultrasound pretreatment, the influence of the input air temperature and sample thickness was significant (*p* < 0.05) through a second-order equation; while for the other pretreatment, these effects were linear and significant (*p* < 0.05). The variations in the dryer efficiency followed a second-order equation for the microwave and control samples; whereas the other pretreatments showed linear significant variation trends (*p* < 0.05).

**Figure 4:**
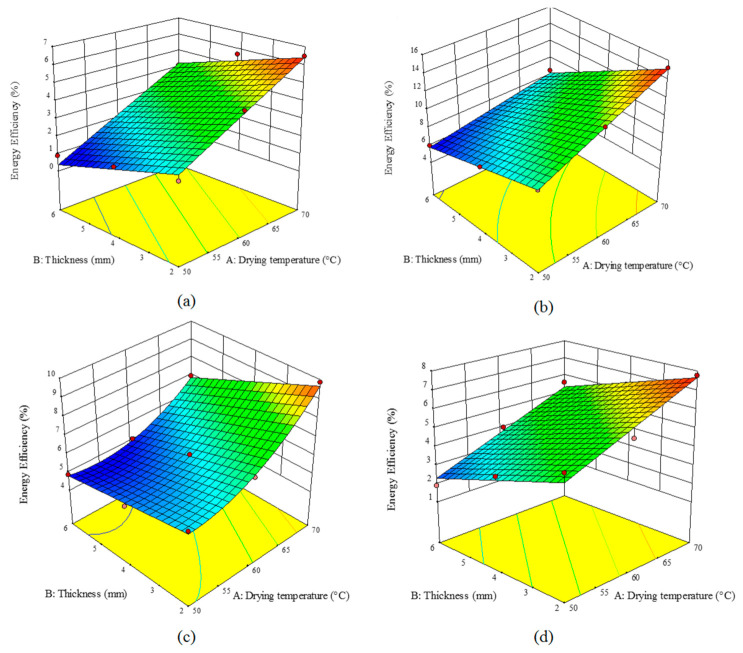
Effect of the drying temperature and sample thickness on the energy efficiency (%) of an HCIR dryer with various pretreatments (**a**) control, (**b**) microwave, (**c**) ultrasound, and (**d**) blanching.

**Figure 5:**
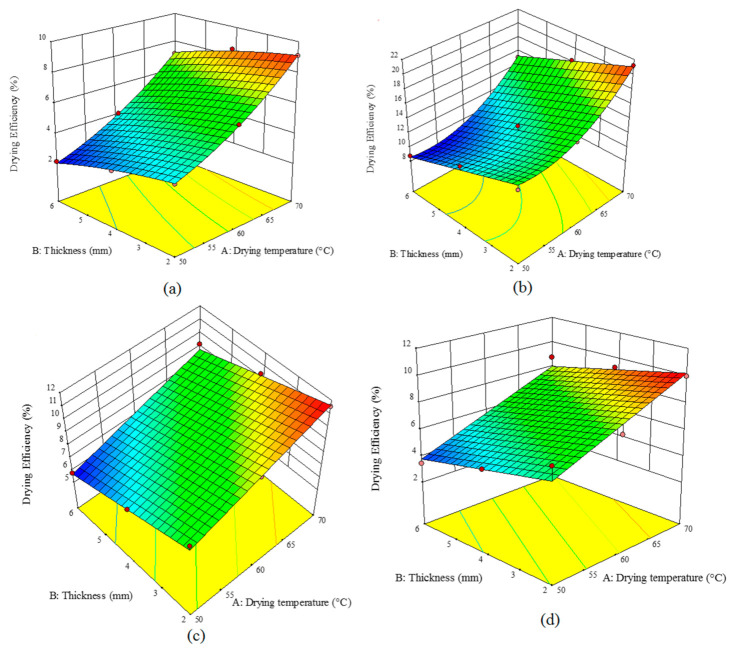
Effect of the drying temperature and sample thickness on the drying efficiency (%) of an HCIR dryer with various pretreatments (**a**) control, (**b**) microwave, (**c**) ultrasound, and (**d**) blanching.

A comparison of Figure 4 and Figure 5 indicated that the elevation of the temperature enhanced the energy and dryer efficiencies. Temperature can augment the rate of moisture removal and hence decline the drying time; therefore, both the efficiencies will show ascending trends with temperature enhancement. With an increase in the sample thickness, the energy and dryer efficiencies declined as the drying time was increased. On the other hand, comparing the studied pretreatments showed that the highest efficiencies can be achieved using the microwave pretreatment (Figure 4b and Figure 5b); while the control samples exhibited the lowest efficiencies (Figure 4a and Figure 5a). Microwave pretreatment destroyed the product texture and accelerated moisture removal. Results have shown that an increase in the drying temperature and a decline in the thickness of the sample can improve both energy and dryer efficiencies. As shown in Figure 4c,d, ultrasonic and blanching pretreatments enhanced the destruction in the product texture, hence no hard layer will be formed during the drying process, and therefore the product will be dried faster. Energy efficiency varied from 0.89% to 6.48% for the controls, 5.99% to 15.23% for the microwave pretreatment, 4.88% to 9.87% for the ultrasound pretreatment, and 1.90% to 7.77% for the blanching pretreatment. The dryer efficiency of the control, microwave, ultrasound, and blanching pretreatments, varied in 2.11–9.11%, 3.45–9.99%, 5.77–11.54%, and 8.74–21.4%, respectively. Other researchers have also shown that various pretreatments can enhance the energy and drying efficiencies [34].

### 3.5. Shrinkage

Table 6 lists the coefficients of the equations obtained by the fitted models for the shrinkage parameter. The air temperature and sample thickness could significantly affect the shrinkage of the samples (*p* < 0.05). Table 6 also shows *R*^2^, adj-*R*^2^, Pre-*R*^2^, and *CV* values. Regarding high *R*^2^ values (above 0.97), the presented model is the best one for predicting the shrinkage level of the samples.

**Figure 6:**
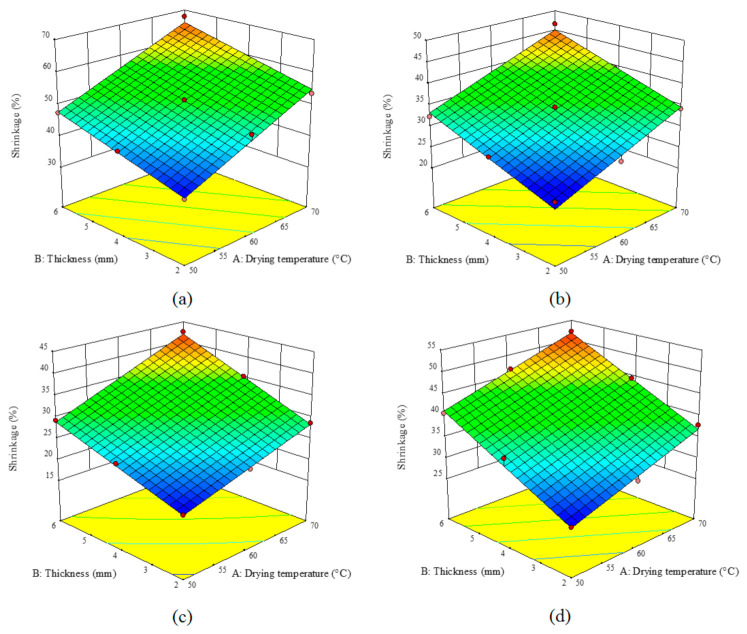
Effect of the drying temperature and sample thickness on the shrinkage (%) of the turnip slices dried under an HCIR dryer with various pretreatments (**a**) control, (**b**) microwave, (**c**) ultrasound, and (**d**) blanching.

Figure 6, shows the influence of the drying air temperature and sample thickness on the shrinkage of the samples pretreated by different methods. As seen, the highest shrinkage can be observed in the control samples while the ultrasound-pretreated samples exhibited the lowest shrinkage (Figure 6a). A comparison of the pretreatments indicated that blanching led to the highest shrinkage as the intercellular water was replaced by air which led to stress in the cell structure, hence the texture failed in maintaining its structure (Figure 6d). As a result, the extracellular structure will collapse resulting in higher shrinkage [29].

The shrinkage increased by increasing the temperature and sample thickness. An increment in the drying temperature enhanced the thermal gradient between the product and the environment, promoting the moisture migration from the internal layers to the sliced layers; this will cause a moisture gradient between the surface and internal layers and hence augment the shrinkage [18]. By drying mushrooms [44] and barley seeds [35] at various temperatures, other researchers also showed an increase in the shrinkage by the temperature elevation. The reason for the increased shrinkage in thicker samples can be explained as follows: a rise in the sample thickness will reduce the water release of the cell and hence decline the stress applied to the cell by the liquid. Such a decline in the stress will enhance the textural shrinkage. The shrinkage of the turnip samples pretreated by microwave (Figure 6b), ultrasound (Figure 6c), and blanching (Figure 6d) method, as well as the controls, varied from 24.28–46.67%, 19.28–42.49%, 26.20–52.21%, and 36.36–67.84%, respectively.

### 3.6. Color Difference (∆E)

As presented in Table 7, the drying air temperature and sample thickness linearly and significantly altered ∆*E* of the dried turnip (*p* < 0.059). *R*^2^, adj-*R*^2^, and Pre-*R*^2^ values of ∆*E* index were above 0.97, above 0.96, and above 0.92. Therefore, the presented equations can well fit the experimental data.

**Figure 7:**
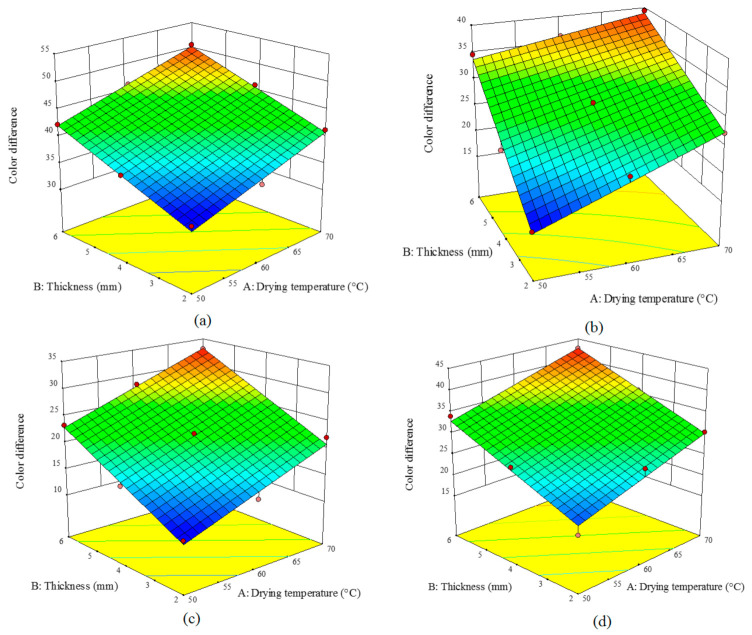
Effect of the drying temperature and sample thickness on the color difference of the turnip slices dried under an HCIR dryer with various pretreatments (**a**) control, (**b**) microwave, (**c**) ultrasound, and (**d**) blanching.

Figure 7 shows the effects of the drying temperature and sample thickness on ∆*E* of the turnip samples dried by an HCIR dryer for different pretreatments. An increase in the temperature and sample thickness enhanced the ∆*E* value since an increase in these two factors implies drying at higher temperatures which will result in browning reactions and an increase of the brunt areas on the sample surface [11]. Similar results were reported on the variations of ∆*E* during drying different products such as almond kernel [45] and cabbage [18].

The highest color variation (∆*E*) was 50.59 and observed in the control samples with the thickness of 6 mm dried at 70 °C (Figure 7a); while the lowest color difference (11.12) was for the ultrasound-treated samples with the thickness of 2 mm dried at 50 °C (Figure 7c). The results indicated that the color indices were closer to the fresh samples when the products were thinner and dried at lower temperatures. According to Figure 7, the studied pretreatments caused some color variations. Similar results were also reported for other agricultural products such as mushrooms [44], star anise [46], cranberry snacks [17], and blackberry [39].

### 3.7. Optimization

Table 8 lists the optimized values of the independent and response variables along with their desirability function based on the desirability index. The optimal independent variables were drying temperature of 70 °C and thickness of 2 mm for all the pretreatments and control samples (accuracy over 0.89). Under this optimal condition, the response variables such as drying time (11.33 min), *SEC* (59.31 MJ/kg), shrinkage (54.87%) and color variation (40.83) were minimized while, *D_eff_* (2.15 × 10^−9^ m^2^/s) energy efficiency (6.64%) and dryer efficiency (9.13) showed their maximal levels. Other researchers also used the RSM method to optimize the drying process of various crops including apricots [45], lavender leaves [25], sunflower seeds [21], and pistachio [47].

### 3.8. ANIFIS

Table 9 presents the results obtained by the ANFIS model to predict the drying time, *D_eff_*, *SEC*, energy and dryer efficiencies, shrinkage, and color variation of the dried turnip samples using an HCIR dryer. To measure the performance of the model, developed equations and two statistical functions, root mean square error (RMSE) and determination coefficient (*R*^2^), were used. In this table the lowest RMSE and highest *R*^2^ are presented. According to Table 7, *R*^2^ of prediction of drying time, *D_eff_*, *SEC*, energy efficiency, dryer efficiency, shrinkage, and color were 0.9965, 0.989, 0.000, 0.9993, 0.9989, and 0.9990, respectively (other pretreatments are shown in Table 9). According to Table 9, it can be concluded that the ANFIS model offered higher accuracy for all the studied parameters as compared with the RSM model. By drying almonds [20] and blackberry [30], the researchers have shown that the ANFIS model can successfully predict the drying properties of the products.

## 4. Conclusions

In this study, drying time, *D_eff_*, *SEC*, energy efficiency, drying efficiency, color, and shrinkage of the turnip samples dried by an HCIR dryer were evaluated under various pretreatments (microwave, ultrasonic, and blanching). The following results were obtained:

- The lowest drying time (40 min), *D_eff_* (1.007 × 10^−9^ m^2^/s), and *SEC* (21.57 Mj/kg) were observed in the microwave pretreatment.
- Energy and dryer efficiencies of 0.89–15.23% and 2.11–21.20% were recorded for the microwave-pretreated samples with a thickness of 2 mm which were dried at 70 °C.
- In the HCIR dryer, *SEC* declined by increasing the temperature and reducing the thickness, microwave power, and blanching temperature; the energy and dryer efficiencies were increased.
- The ultrasonic pretreatment led to the lowest shrinkage (19.28%) and color variation (11.12) moreover, an increase in the temperature and sample thickness enhanced the shrinkage and color variations for all the pretreatments.
- The optimal condition for the lowest *SEC* and the highest energy and dryer efficiencies involved the air temperature of 70 °C and sample thickness of 2 mm which led to the desirability of over 89% for all the pretreatments.
- A comparison of the parameter prediction by RSM and ANFIS models indicated that the RSM model exhibited very good performance in modeling and optimizing the process; while the ANFIS method did not have this capability. ANFIS, however, showed better performance in predicting the dependent variables.

This study provides an in-depth understanding of the drying kinetics, and energy consumption, energy efficiency and quality properties (shrinkage and color) of HCIR drying process with four pretreatments, which will be helpful for the selection of pretreatment methods in the turnip industry.

## Figures and Tables

**Table 1:** Boundary conditions and the independent and dependent variables.

Variables	Goal	Lower Limits	Upper Limits	Importance
Drying air temperature (°C)	In range	50	70	5
Sample thickness (mm)	In range	2	6	5
Drying time (min)	Minimum	40	250	5
Effective moisture diffusivity (m^2^/s)	Maximum	1.01 × 10^−9^	8.11 × 10^−9^	5
*SEC* (Mj/kg)	Minimum	21.57596	168.98	5
Energy efficiency (%)	Maximum	0.89	15.23	5
Drying efficiency (%)	Maximum	2.11	21.2	5
Color difference	Minimum	11.12	50.59	5
Shrinkage (%)	Minimum	19.28	67.84	5

**Table 2:** Response surface method (RSM) modeling results for predicting the drying time under a hybrid convective-infrared (HCIR) dryer with various pretreatments.

Pretreatment	Equation	*R* ^2^	Adj *R*^2^	Pred *R*^2^	*CV* (%)
Control	348.33 − 3.75 × A + 13.75 × B	0.9830	0.9773	0.9567	3.43
Microwave	87.77 − 0.833 × A + 0.40 × B − 0.50 × A × B	0.9821	0.9713	0.9358	6.78
Ultrasonic	270.55 − 2.91 × A + 6.66 × B	0.9944	0.9925	0.9862	1.97
Blanching	999.44 − 25.5 × A − 12.91 × B + 0.18 × A^2^ + 2.70 × B^2^	0.9924	0.9847	0.9613	3.14

A: Drying temperature (°C); B: Thickness (mm). *R*^2^: determination coefficient and *CV*: Coefficient of variation.

**Table 3:** Response surface method (RSM) modeling for predicting effective moisture diffusivity coefficient (*D_eff_*) under a hybrid convective-infrared (HCIR) dryer with various pretreatments.

Pretreatment	Equation	*R* ^2^	Adj *R*^2^	Pred *R*^2^	*CV* (%)
Control	−2.86 × 10^−10^ + 3.85 × 10^−11^×A + 1.26 × 10^−10^ × B	0.9447	0.9263	0.8776	7.34
Microwave	−4.03 × 10^−9^ + 1.82 × 10^−10^ × A −6.14 × 10^−10^ × B	0.9496	0.9328	0.8674	11.40
Ultrasonic	−7.51 × 10^−10^ + 6.38 × 10^−11^ × A −1.84 × 10^−10^ × B	0.8594	0.8065	0.6041	12.99
Blanching	−7.34 × 10^−10^ + 5.16 × 10^−11^ × A −1.08 × 10^−10^ × B	0.8996	0.8661	0.7817	9.71

A: Drying temperature (°C); B: Thickness (mm). *R*^2^: determination coefficient and *CV*: Coefficient of variation.

**Table 4:** Modeling results by the use of RSM for prediction of specific energy consumption (*SEC*) under an HCIR dryer with various pretreatments.

Pretreatment	Equation	*R* ^2^	Adj *R*^2^	Pred *R*^2^	*CV* (%)
Control	85.75 − 0.60 × A + 45.78 × B − 0.53 × A × B	0.9909	0.9853	0.9830	4.05
Microwave	52.40 − 0.56 × A + 18.83 × B − 0.20 × A × B	0.9954	0.9926	0.9777	3.25
Ultrasonic	132.39 − 1.63 × A + 9.32 × B	0.9329	0.9106	0.8484	9.29
Blanching	44.38 − 0.06 × A + 33.97 × B − 0.46 × A × B	0.9866	0.9786	0.9432	4.54

A: Drying temperature (°C); B: Thickness (mm).

**Table 5:** RSM modeling of the energy and dryer efficiencies under an HCIR dryer with different pretreatments.

Variable	Pretreatment	Equation	*R* ^2^	Adj. *R*^2^	Pred. *R*^2^	*CV* (%)
$\eta_{e}$	Control	−6.04 + 0.19 × A − 0.54 × B	0.9706	0.9608	0.9271	11.39
	Microwave	−7.97 + 0.37×A + 1.26B − 0.04 × A × B	0.9951	0.9921	0.9721	2.73
	Ultrasonic	26.00 − 0.82 × A + 0.73B − 0.02 × A × B + 0.008 × A^2^	0.9893	0.9787	0.9276	3.58
	Blanching	−1.75 + 0.15 × A − 0.58 × B	0.9618	0.9491	0.8999	7.63
$\eta_{d}$	Control	11.70 − 0.44 × A − 0.11 × B + 5.85 × A^2^ − 0.04 × B^2^	0.9991	0.9981	0.9952	1.94
	Microwave	82.80 − 2.49 × A − 1.42 × B + 0.02 × B^2^	0.9897	0.9834	0.9665	3.69
	Ultrasonic	2.11 + 0.15 × A − 0.70 × B	0.9790	0.9720	0.9520	3.55
	Blanching	−3.57 + 0.21 × A − 0.54 × B	0.9314	0.9085	0.8067	9.24

A: Drying temperature (°C); B: Thickness (mm). Energy ($\eta_{e}$) and dryer ($\eta_{d}$) efficiency.

**Table 6:** RSM modeling for predicting shrinkage of the turnip samples under an HCIR dryer with different pretreatments.

Pretreatment	Equation	*R* ^2^	Adj. *R*^2^	Pred. *R*^2^	*CV* (%)
Control	−14.24 + 0.90 × A + 2.75 × B	0.9821	0.9761	0.9547	2.80
Microwave	−12.17 + 0.59A + 2.63 × B	0.9768	0.9691	0.9367	2.09
Ultrasonic	−3.99 + 0.36×A + 0.07 × B + 0.04 × A × B	0.9951	0.9922	0.9809	3.60
Blanching	−8.41 + 0.53 × A + 3.76 × B	0.9840	0.9787	0.9692	3.02

A: Drying temperature (°C); B: Thickness (mm).

**Table 7:** RSM modeling for predicting color difference (∆*E*) of the samples dried under an HCIR dryer with different pretreatments.

Pretreatment	Equation	*R* ^2^	Adj. *R*^2^	Pred. *R*^2^	*CV* (%)
Control	+7.31 + 0.41 × A + 2.3 × 6B	0.9861	0.9815	0.9706	1.79
Microwave	−33.93 + 0.79 × A + 9.08 × B − 0.08 × A × B	0.9880	0.9808	0.9580	3.72
Ultrasonic	−21.38 + 0.51×A + 3.09 × B	0.9871	0.9829	0.9686	4.21
Blanching	−10.61 + 0.49 × A + 3.15 × B	0.9720	0.9627	0.9269	4.31

A: Drying temperature (°C); B: Thickness (mm).

**Table 8:** Optimization of the response parameters for turnip drying under an HCIR dryer with different pretreatments by RSM.

Pretreatment	Air Temperature (°C)	Thickness (mm)	Time (min)	*D_eff_* (m^2^/s)	*SEC* (MJ/kg)	$\eta_{e}$ (%)	$\eta_{d}$ (%)	Shrinkage (%)	Color Difference	Desirability
Control	70	2	113.33	2.15 × 10^−9^	59.31	6.40	9.13	54.87	40.83	0.896
Microwave	70	2	39.44	7.49 × 10^−9^	21.59	15.12	21.06	34.62	27.40	0.893
Ultrasonic	70	2	79.72	3.34 × 10^−9^	36.59	9.67	11.64	28.43	20.75	0.892
Blanching	70	2	97.77	2.66 × 10^−9^	53.30	7.66	10.20	36.87	30.07	0.911

**Table 9:** Prediction of the response parameters for turnip drying under an HCIR dryer with different pretreatments by ANFIS.

Pretreatment	Time (min)		*Deff* (m^2^/s)		*SEC* (Mj/kg)		$\eta_{e}$ (%)		$\eta_{d}$ (%)		Shrinkage (%)		Color	
	*R* ^2^	MSE	*R* ^2^	MSE	*R* ^2^	MSE	*R* ^2^	MSE	*R* ^2^	MSE	*R* ^2^	MSE	*R* ^2^	MSE
Control	0.9975	0.0012	0.9690	0.0059	0.9995	0.0002	0.9859	0.0012	0.9994	0.0002	0.9968	0.0009	0.9979	0.0008
Microwave	0.9965	0.0019	0.9890	0.0022	0.9990	0.0004	0.9993	0.0004	0.9989	0.0004	0.9869	0.0020	0.9990	0.0004
Ultrasonic	0.9990	0.0004	0.9797	0.0048	0.9805	0.0017	0.9896	0.00011	0.9939	0.0010	0.9996	0.0002	0.9990	0.0004
Blanching	0.9980	0.0008	0.9708	0.0054	0.9989	0.0004	0.9979	0.0008	0.9928	0.0011	0.9979	0.0008	0.9988	0.0004

## Data Availability

Data for this research will available.

## Nomenclatures

$D_{eff}$	Effective moisture diffusion coefficient (m^2^/s)
$E_{t}$	Total energy input to dryer (MJ)
EU	Total energy consumption
$E_{evap}$	Energy consumed to evaporate moisture from drying samples (kJ)
$E_{heating}$	Energy for the material heating (kJ)
*M*	weight of the sample (g)
*Mf*	weight of pycnometer (g),
$M_{b}$	Initial moisture content (kg _water_/kg _dry matter_)
$M_{e}$	Equilibrium moisture content (kg _water_/kg _dry matter_)
$M_{t}$	Moisture content at any time (kg _water_/kg _dry matter_)
MR	Moisture ratio
*Msf*	weight of the toluene for filling the pycnometer (g)
*Mt+s*	weight of pycnometer plus the weights of the sample and toluene (g)
N	Number of data values
*R* ^2^	determination coefficient
$S_{b}$	Shrinkage (%)
SEC	Specific energy consumption (MJ/kg)
$S_{k}$	Predict data
t	Drying time (min)
$T_{k}$	Experimental data
$T_{m}$	average predicted values
*Vf*	pycnometer volume (cm^3^)
$V_{o}$	Final volume (cm^3^)
$V_{t}$	Initial volume (cm^3^)
ΔE	Total color change
$\Delta L^{*}$,$\Delta b^{*}$,$\Delta a^{*}$	Differences between the color of the fresh and dried sample
ρs	density of toluene (0.87 g/cm^3^ at 20 °C)
$\eta_{d}$	Drying efficiency (%)
$\eta_{e}$	Energy efficiency (%)
$S_{a}$	Shrinkage (cm^3^)
